# Enhancing Spatial Ability Assessment: Integrating Problem-Solving Strategies in Object Assembly Tasks Using Multimodal Joint-Hierarchical Cognitive Diagnosis Modeling

**DOI:** 10.3390/jintelligence13030030

**Published:** 2025-03-05

**Authors:** Jujia Li, Kaiwen Man, Joni M. Lakin

**Affiliations:** Department of Educational Studies in Psychology, Research Methodology and Counseling, College of Education, University of Alabama, Tuscaloosa, AL 35487, USA; kman@ua.edu (K.M.); jlakin@ua.edu (J.M.L.)

**Keywords:** spatial reasoning ability, response time, eye tracking, MJ-DINA model

## Abstract

We proposed a novel approach to investigate how problem-solving strategies, identified using response time and eye-tracking data, can impact individuals’ performance on the Object Assembly (OA) task. To conduct an integrated assessment of spatial reasoning ability and problem-solving strategy, we applied the Multimodal Joint-Hierarchical Cognitive Diagnosis Model (MJ-DINA) to analyze the performance of young students (aged 6 to 14) on 17 OA items. The MJ-DINA model consists of three sub-models: a Deterministic Inputs, Noisy “and” Gate (DINA) model for estimating spatial ability, a lognormal RT model for response time, and a Bayesian Negative Binomial (BNF) model for fixation counts. In the DINA model, we estimated five spatial cognitive attributes aligned with problem-solving processes: encoding, falsification, mental rotation, mental displacement, and intractability recognition. Our model fits the data adequately, with Gelman–Rubin convergence statistics near 1.00 and posterior predictive *p*-values between 0.05 and 0.95 for the DINA, Log RT, and BNF sub-models, indicating reliable parameter estimation. Our findings indicate that individuals with faster processing speeds and fewer fixation counts, which we label Reflective-Scanner, outperformed the other three identified problem-solving strategy groups. Specifically, sufficient eye movement was a key factor contributing to better performance on spatial reasoning tasks. Additionally, the most effective method for improving individuals’ spatial task performance was training them to master the falsification attribute. This research offers valuable implications for developing tailored teaching methods to improve individuals’ spatial ability, depending on various problem-solving strategies.

## 1. Introduction

Spatial ability is a critical cognitive skill for reasoning and problem-solving, especially for STEM education. Despite its importance, scholars debate its definition and structure, with recent research highlighting its role as a distinct cognitive process. Spatial ability has been a prominent topic in cognitive research for decades, attracting plenty of attention from scholars in intelligence assessment, educational psychology, and cognitive psychology. Spatial ability plays a vital role in everyday tasks and cognitive processes, especially in mathematical and scientific reasoning (Nuttall et al. 2005; Reilly and Neumann 2013; Serbin et al. 1990; Wai et al. 2009). Wai et al. (2009) emphasized the significance of spatial ability in developing skills in STEM and its predictive power in talent identification. Carroll (1993) and Halpern (2000) addressed that there is a robust correlation between spatial ability and mathematical achievement, which may offer a pathway for improving instructional methodologies and supporting STEM education (see also Hawes and Ansari 2020; Lohman 1996; Shepard 1978). Furthermore, spatial ability is regarded as a fundamental skill for geometrical reasoning (Ramful et al. 2017).

Despite its importance, there is a lack of consensus regarding the definition, categorization, and assessment of spatial ability. Spatial ability has been defined as the ability to generate, retain, retrieve, and transform well-structured images (Lohman 1996; Ramful et al. 2017). In the past fifty years, research has revealed a growing intricacy in its conceptualization, from a job-specific skill to a key component of advanced reasoning and creativity.

Nevertheless, these abilities are usually overlooked in discussions over human intelligence, typically linked to practical skills rather than abstract reasoning (Lohman 1996; Sternberg and Kaufman 2011; Wai 2022; Wai and Worrell 2016). In the last two decades, numerous researchers have consistently endorsed the idea that, according to neuroimaging and educational psychology studies, spatial ability is a distinct brain activation and an independent cognitive process (Höffler 2010; Uttal et al. 2013).

### 1.1. Facets of Spatial Ability

Based on these fundamental understandings of spatial ability, we conducted additional explorations of the specific spatial attributes that support effective cognitive processes in spatial reasoning tasks. These attributes provide a profound understanding of the frameworks that influence spatial ability.

Much like other broad cognitive abilities, spatial ability has demonstrated both a strong general factor as well as some number of specific abilities. Despite consensus on the multidimensional nature of spatial ability, the exact number and content of each of its component factors remain debated (Ramful et al. 2017; Yilmaz 2009). A major challenge to distinguishing remarkably comparable spatial attributes. Furthermore, these components often exhibit moderate to strong correlations, with many depending on visual working memory (Höffler 2010; Miyake et al. 2001).

The categorization of spatial ability attributes originated from early psychometric work by Thurstone and Thurstone (1941) and continued with factor analyses by Lohman (1996) and McGee (1979). In these studies, the researchers concurred on important elements of spatial ability, such as visualization and orientation, which set a base for later categorizations. McGee (1979) addressed one of the initial and most powerful classifications, which includes five parts: spatial perception, spatial visualization, mental rotation, spatial relations, and spatial orientation. Other commonly reported subdivisions of the broad group spatial factor were visualization, spatial orientation, and speeded rotation (Anderson et al. 2005; Linn and Petersen 1985; Lohman 1996; Ramful et al. 2017).

Other researchers have focused on the cognitive processes required by an individual while successfully solving spatial tasks (Table 1). Cooper and Shepard (1973) proposed a four-stage model for solving mental rotation tasks, involving encoding stimuli, mental rotation, confirmation, and motor response. Pellegrino et al. (1985) then addressed their three-stage model, which included falsification, mental rotation, and verification. While Cooper and Shepard (1973) and Pellegrino et al. (1985) treated confirmation/verification as a single step, which cannot fully capture the complexities of object assembly tasks, Embretson and Gorin (2001) refined the confirmation process into three distinct sub-attributes: rotation, displacement, and intractability. Then, confirmation was further divided into confirmation I (the key distractors, including rotation and displacement) and confirmation II (the non-falsifiable distractor). Compared to spatial ability factor analyses, which are more focused on spatial ability itself, research on the cognitive processes focuses on the capabilities required to solve specific spatial problems, providing a more granular understanding of spatial ability.

To achieve a satisfactory performance in a spatial test, a person should manifest strong spatial ability by using effective strategies for solving spatial problems. In the next section, we concentrated on the categorization of problem-solving types in a spatial assessment. This sets the stage for examining how spatial problem-solving strategies interact with one another (Wang and Chiew 2010).

### 1.2. Problem-Solving Strategy

In spatial tests, some researchers found that performance may not solely depend on spatial reasoning abilities but also on problem-solving strategies (Kanamori and Yagi 2002; Kozhevnikov et al. 2002). Examinees can use a variety of strategies and cognitive processing styles when engaging with any assessments (Bornstein 2011; Gorin 2006). Problem-solving strategies and cognitive styles are specifically defined as the tendencies that individuals have while responding to test tasks that can affect either their accuracy or their speed of responding (Sternberg and Grigorenko 1997).

Two continuums of cognitive styles have been found to relate to examinee performance in recent research on other cognitive tasks (math knowledge, Zhan et al. 2022; Raven’s Progressive Matrices, Liu et al. 2023). One contrast is reflective vs. impulsive cognitive styles (Shepard and Metzler 1971). Reflective individuals take their time to deliberate before deciding, leading to higher accuracy, while impulsive individuals make faster decisions, often with more errors. Another useful contrast is between scanner and focuser styles (Nazareth et al. 2019), based on the breadth and intensity of their visual engagement (Gardner et al. 1959). Scanners exhibit a broad attention range with low intensity during problem-solving, whereas focusers show a narrow range yet a highly intensely concentrated attention span, which may reflect how individuals approach complex spatial tasks differently.

This categorization provides a crucial dimension for understanding the selection of diverse problem-solving strategies in spatial reasoning. Unkelbach (2006) observed significant differences in individuals’ cognitive fluency patterns when they encounter ease or difficulty in solving mental rotation tasks. Those with higher cognitive fluency may be more likely to adopt both reflective and scanner styles, as they can efficiently process spatial information and explore multiple solutions. A possible reason is that cognitive fluency allows for rapid comparison and mental manipulation of spatial information, facilitating both the broad attentional sweep of scanners and the deliberate processing of reflective individuals.

By integrating processing speed cognitive styles (reflective and impulsive) with visual engagement styles (scanner or focuser), participants can be categorized into four groups: Impulsive Focusers, Impulsive Scanners, Reflective Focusers, and Reflective-Scanners. This categorization integrates response time and fixation counts, providing a foundation for assessing and observing individuals’ performance in spatial tasks based on their strategy selection.

### 1.3. Traditional and Objective Measures of Problem-Solving

Traditional approaches to identifying problem-solving strategies include self-reports or strategy questionnaires, but these methods have some drawbacks, such as biases and errors of estimation (Nazareth et al. 2019). These methods are limited in their accuracy when capturing the complex processes individuals use in spatial tasks. Self-assessment of styles is a key reason that learning styles have been resoundingly ineffective in instructional design (Kirschner 2017).

To address these limitations, objective measures like response times and accuracy can provide a more reliable and nuanced understanding of how individuals approach spatial tasks. By analyzing these metrics, researchers can better understand whether individuals are employing a reflective or impulsive cognitive style, marking a significant advancement in the study of problem-solving strategies, enabling a deeper comprehension of strategies impacting performance in spatial tasks (Man et al. 2022; Man and Harring 2019; Zhan et al. 2022).

Building on this, researchers have employed Response Time (RT) to distinguish differences in problem-solving strategies (Heil and Jansen-Osmann 2008; Kail et al. 1979; Kanamori and Yagi 2002; Shepard and Metzler 1971). However, response time alone cannot fully reflect participants’ engagement in spatial tasks, as it provides only an overall measure of cognitive duration without detailing the proportion of time allocated to specific cognitive processes. Eye-tracking technology, particularly fixation count (the frequency of gaze movements), provides additional insight into how individuals allocate their attention and manage their time during tasks (Nazareth et al. 2019). Early studies used eye-tracking to establish the foundation for examining cognitive processes in mental rotation by recording eye movements (Just and Carpenter 1976).

Eye-tracking data can reveal various types of information about participant visual scanning behaviors, especially within spatial tasks, via indicators such as fixation duration, fixation count, visit duration, and visit count (Nazareth et al. 2019). The correlation between fixation counts and visual engagement enables researchers to classify individuals as either Scanners or Focusers, based on the breadth and intensity of their visual engagement (Gardner et al. 1959).

### 1.4. Multimodal Assessments

Traditional assessments of spatial ability often focus exclusively on response accuracy without processing data due to most psychometrics models’ limitations. To address this limitation, recently, multimodal has dramatically developed as a powerful tool for researchers to analyze complex data structures with multiple sources, especially process data in cognitive assessment. To achieve a holistic and simultaneous analysis of OA test responses, response times, and fixation counts, this study utilizes the Multimodal Joint-Hierarchical Cognitive Diagnosis Model (MJ-DINA). The MJ-DINA model integrates three sub-models: the higher-order deterministic input, the noisy “and” gate (DINA) model (De La Torre 2011) for estimating general spatial ability and specific attributes, the lognormal RT model (Van der Linden 2006) for measuring response time, and the negative binomial fixation (NBF) model (Man and Harring 2019) for analyzing fixation counts. Zhan et al. (2022) proved that the MJ-DINA can improve parameter recovery and classification accuracy, particularly when correlations exist between person parameters through a simulation study. Before introducing the three sub-models, we will first explain the MJ-DINA model. Since the MJ-DINA model is a comparatively complex model with three sub-models, we will provide a detailed introduction to it in a separate Model section.

### 1.5. Research Questions

Our approach is threefold, aiming to provide readers with innovative insights into spatial ability assessment. First, we analyze the cognitive processes and psychometrics involved in OA tasks. Next, we incorporate response time and eye-tracking data to investigate problem-solving strategies. Lastly, we investigate how different problem-solving strategies affect individual variations in spatial test performance, contributing a new perspective to future spatial reasoning ability research. The research questions are as follows:

1. How well does the MJ-DINA model fit the observed data on spatial reasoning performance, response times, and fixation counts?

2. Which problem-solving strategy is most strongly associated with better performance on object assembly (OA) tasks?

3. How do spatial ability attributes, as specified in the DINA model, interact with problem-solving strategies to influence performance in OA tasks?

## 2. Materials and Methods

### 2.1. Instrument

This study employs the Object Assembly (OA) test to assess individuals’ spatial reasoning abilities. This test format was initially known as the Minnesota Paper Form Board Test (MPFBT; Quasha and Likert 1937) and challenged examinees to assemble disparate pieces into a coherent objects. According to the classification of spatial tasks by Zhan et al. (2018), the OA task primarily evaluates spatial skills in two-dimensional rotation and visualization, as delineated by Lohman (1979). Additionally, the OA test is ideal for studying eye movements, providing insights into how people visually process spatial information and the cognitive strategies they employ. Furthermore, it reveals insights into various problem-solving strategies, showing an individual’s adaptability and flexibility in solving spatial tasks.

**Figure 1:**
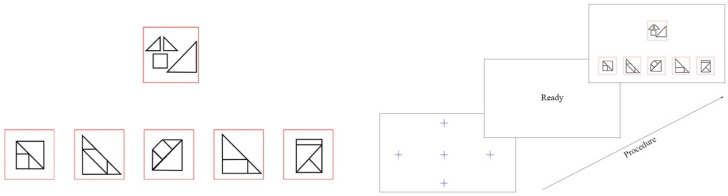
Example of An Object Assembly Item with Test Procedure.

In addition, items used in this study are inspired by the research of Embretson and Gorin (2001). In the OA task, participants must recognize the pieces presented in the stem and compare them with five options to determine which one accurately represents the shape that can be assembled from the pieces in the stem (Figure 1). After conducting pilot testing, we selected fifteen items that demonstrated diverse levels of difficulty and strong discrimination statistics.

### 2.2. Sample

After excluding data from subjects who failed to complete the eye tracking tasks (usually due to calibration failures), a total of *N* = 50 children aged 6 to 14 years (*M* = 10) participated and completed this computer-based OA test. To ensure the quality of eye-tracking data, all recruited participants had normal or corrected-to-normal vision. The sample consisted of 46% male (*n* = 23) and 54% female (*n* = 25) participants, exhibiting a relatively balanced gender distribution. This study was conducted under the oversight of the authors’ institution. Parents or legal guardians of children were contacted using a participant registry for a university research center and invited to have their children participate in this study. Prior to the study, interested parents received a survey and consent form. Students also assented to participation after completing the OA assessment, and participants’ parents received a $50 gift card.

### 2.3. Procedure

The research procedure involves solving OA tasks on a computer, with eye movements monitored by an eye-tracker, the Eyelink Portable Duo by SR Research (sr-research.com, accessed on 1 May 2023), configured for monocular tracking at a frequency of 500 Hz. The session lasted about 90 min, including breaks as needed. It consisted of five components: a 5 min assent and eye-tracking calibration, a 10 min OA subtest (with eye-tracking), a 20 min Think Aloud interview, a 30 min spatial test battery (without eye-tracking), and a 10 min survey. This study specifically focused on the first 10 min OA task, ensuring most young participants maintained consistent attention. Each participant was tested one-on-one in a quiet environment. For eye tracking, participants sat approximately 60 cm from the display, with the aid of a chin rest to stabilize the head position. A research assistant managed the eye-tracking system via a monitor laptop, ensuring the experiment’s uninterrupted execution. The Eyelink system recorded eye movements as participants engaged in spatial puzzles and concluded with equipment cleaning and a break.

Figure 1 displays a sample item from the OA test, which includes a stem, positioned at the top center of the screen, with five options arranged below it. The task for participants is to select the option that can be formed using the pieces presented in the stem. To the right of Figure 1, the test sequence is outlined, beginning with a five-point calibration technique to fine-tune the participant’s gaze coordinates before starting the response sequence. This was followed by a preliminary “ready” indicator at the center of the screen that required the participant’s gaze before moving on to the next item via mouse click. After selecting an answer choice by clicking one of the options on the screen, participants then repeated this process for each subsequent question until the test was completed.

### 2.4. Model

To answer the research questions and achieve a holistic and simultaneous analysis of OA test responses, response times, and fixation counts, this study utilizes the Multimodal Joint-Hierarchical Cognitive Diagnosis Model (MJ-DINA). The Multimodal Joint-Hierarchical DINA (MJ-DINA) model is an advanced model initially addressed and tested by Zhan et al. (2022) and his colleagues. This model extends the standard DINA model by integrating hierarchical and multimodal data structures, including response accuracy (RA), response time (RT), and fixation counts (FC). Zhan et al. (2022) and colleagues have used two simulation studies to test the feasibility of the MJ-DINA model, suggesting that the parameters of the model can be well recovered. It is designed for evaluating educational assessments and cognitive process diagnoses, especially in spatial reasoning tests. Integrating RT and FX data to RA not only allows us to directly look at how the multiple variables are interacted, but it also improves the precision of estimating the parameters in the different measurement models (Man and Harring 2019; Zhan et al. 2022).

**Figure 2:**
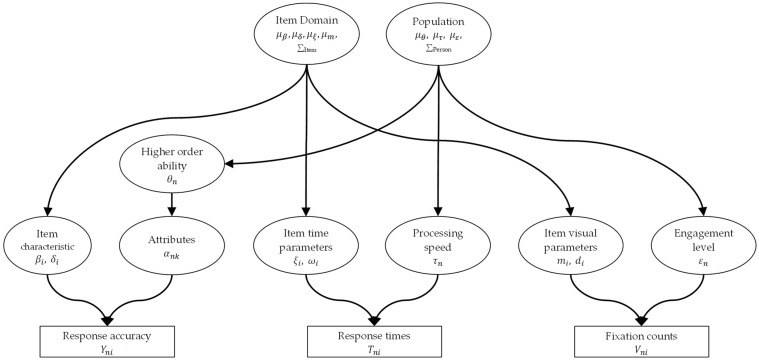
A graphical structure of Multimodal Joint-Hierarchical DINA (MJ-DINA) Model. In the higher-order DINA model, $\beta_{i}$ and $\delta_{i}$ are item parameters, $\theta_{n}$ is person n’s latent ability, $\alpha_{nk}$ represents attributes, and $Y_{ni}$ is accuracy. In the lognormal RT model, $\omega_{i}$ is the reciprocal of the error standard deviation, $\xi_{i}$ is time-intensity, $\tau_{n}$ is processing speed, and $T_{ni}$ is response time. In the NBF model, $m_{i}$ is visual intensity, $d_{i}$ is visual discrimination, $\epsilon_{n}$ is visual engagement, and $V_{ni}$ is fixation count. Means are $\mu_{\beta },\mu_{\delta },\mu_{\xi },\mu_{m}$ (item parameters) and $\mu_{\theta },\mu_{\tau },\mu_{\varepsilon }$ (person parameters).

In the MJ-DINA model, variables such as $Y_{ni}$ (response accuracy), $T_{ni}$ (log-transformed response time), and $V_{ni}$ (fixation counts) are modeled individually at the first level; at the second level, two latent structures for variance and covariance are incorporated to account for correlations among item and person parameters respectively, shown as Figure 2:

The MJ-DINA model integrates the DINA model for RA, a lognormal RT model for response times, and a Negative Binomial Fixation model for FCs. Both Item parameters (e.g., $\mu_{\beta },\mu_{\delta },\mu_{\xi },\mu_{m}$) and person parameters (e.g., $\mu_{\theta },\mu_{\tau },\mu_{\varepsilon }$) in the MJ-DINA model are presumed to follow two separate multivariate normal distributions:

(1) $$\Psi_{i}=\left(\begin{array}{c} \beta_{i}\\ \delta_{i}\\ \xi_{i}\\ m_{i} \end{array}\right)∼MVN\left(\left(\begin{array}{c} \mu_{\beta }\\ \mu_{\delta }\\ \mu_{\xi }\\ \mu_{m} \end{array}\right),\sum_{item}\right),\sum_{item}=\left(\begin{array}{ccc} \alpha_{\beta }^{2} & \cdots & \sigma_{\beta m}\\ \vdots & \ddots & \vdots \\ \sigma_{m\beta } & \cdots & \alpha_{m}^{2} \end{array}\right),$$

where $\mu_{item}=(\mu_{\beta },\mu_{\delta },\mu_{\xi },\mu_{m})$ is the mean vector of item parameters and $\Sigma_{item}$ is the variance and covariance matrix of item parameters. According to Man et al. (2022) and Zhan et al. (2018), to simplify computation, our model only included four item-side parameters whose interrelationships can be easily explained in multivariate normal distribution, and other item parameters (e.g., $\omega_{i},\;\xi_{i}$), assumed to be independently distributed, were not included in (Van der Linden 2006; Man et al. 2022).

(2) $$\Theta_{n}=\left(\begin{array}{c} \theta_{n}\\ \tau_{n}\\ \varepsilon_{n} \end{array}\right)∼MVN\left(\left(\begin{array}{c} \mu_{\theta }\\ \mu_{\tau }\\ \mu_{\varepsilon } \end{array}\right),\sum_{person}\right),\sum_{person}=\left(\begin{array}{ccc} \sigma_{\theta }^{2} & & \\ \sigma_{\tau \theta } & \sigma_{\tau }^{2} & \\ \sigma_{\varepsilon \theta } & \sigma_{\varepsilon \tau } & \sigma_{\varepsilon }^{2} \end{array}\right),$$

where $\mu_{person}=(\mu_{\theta },\mu_{\tau },\mu_{\varepsilon })$ is the mean vector of person parameters and $\Sigma_{person}$ is the covariance of matrix of all person parameters ($\theta_{n}$, $\tau_{n}$, and $\varepsilon_{n}$) from three sub-models.

#### 2.4.1. The Higher-Order DINA Model

The deterministic input, noisy “and” gate model (DINA) evaluates individuals’ observed responses (e.g., correct or incorrect answers in a test) by deconstructing them into a collection of underlying attributes or skills (Haertel 1989). It is a conjunctive model, in which examinees are not expected to answer an item correctly unless they master all required attributes. This method provides detailed insight into their cognitive capabilities. Zhan et al. (2022) addressed the equation associated with the DINA model as above:

(3) $$\log it(P(Y_{ni}=1))=\beta_{i}+\delta_{i}\prod_{k=1}^{K}\alpha_{nk}^{q_{ik}},$$

in which logit(x)=log(x/(1−x)); $\alpha_{n}={\left(\alpha_{n1},\ldots ,\alpha_{nK}\right)}^{\prime }$ denotes the nth participants’ attributes, $\alpha_{nk}\epsilon \{0,1\}$, where $\alpha_{nk}=1$ if person n masters attribute *k* (*k* = 1, 2, …, *K*) and $\alpha_{nk}=0$ if not, where *K* is the number of required attributes in a test.

(4) $$\beta_{i}=\log(\frac{g_{i}}{1-g_{i}}),$$

in which, $\beta_{i}$ is the intercept parameter for item *i* and $g_{i}$ is common guessing.

(5) $$\delta_{i}=\log(\frac{1-s_{i}}{s_{i}})-\log(\frac{g_{i}}{1-g_{i}}),$$

in which, $\delta_{i}$ is the interaction parameter for item *i* and $s_{i}$ is slipping.

To accommodate the binary nature of “$\alpha_{nK}$” in joint-hierarchical modeling, we employed a higher-order latent structural model based on Equation (3), following the approach of Zhan et al. (2018) and De La Torre and Douglas (2004), which is shown below:

(6) $$\log it(P(\alpha_{nk}=1))=\gamma_{k}\theta_{n}-\lambda_{k},$$

in which, $\theta_{n}$ is the higher order latent ability of person n, following the standard normal distribution for model identification; $\gamma_{k}$ and $\lambda_{k}$ are the slope and difficulty parameters for attribute *k*, respectively.

In the CDM framework, the most critical part is to carefully design a Q-matrix, the relationship between spatial attributes and items. Before creating Q-matrix, we employed Embretson and Gorin’s (2001) five-attribute spatial cognitive model to define the rule for creating Q-matrix (see Table 2). To establish validity, we performed expert reviews and a pilot study, ensuring that the tasks measure distinct spatial cognitive attributes.

Table 2 shows the definition of specific spatial cognitive process (e.g., encoding, falsification, mental rotation, displacement, and intractability recognition), rather than simply listing sources of difficulty. Specifically, encoding difficulty is affected by the number of pieces and the complexity of the pieces in the stem, such as the number of edges, curves, and clear verbal labels (e.g., circle, triangle). More complex and ambiguous shapes lead to higher cognitive load at this stage. The falsification stage is to eliminate response alternatives with grossly inappropriate features, such as the wrong number of pieces, relative sizes, and shapes. The ability to quickly identify these mismatches is key to reducing item difficulty and an individual’s cognitive effort. In the confirmation stage, we propose three cognitive processes related to rotation, displacement, and intractability. The rotation process requires mental manipulation and visual comparison. More rotated pieces in alternatives make this stage more challenging. The displacement stage represents the difficulty of identifying displaced pieces in the response options. More pieces in alternatives are moved to different locations, making it more difficult to compare the stem and alternatives. The difficulty of confirming the intractability of alternatives involves assessing the number of comparison cycles needed to find a mismatch and the proportion of pieces mismatched by small angular disparities. More subtle mismatches make disconfirmation more challenging.

This study defines a Q-matrix derived from the five essential spatial cognitive attributes outlined in Table 3. Each of the seventeen items was assigned a value of either 1 or 0, signifying the necessity of mastering a specific spatial attribute for participants to answer the item correctly. The Q-matrix utilized in this research is outlined below:

The coding of these items was determined through a consensus among multiple experts to ensure a more reliable and objective classification. In Table 3, encoding (EN) in spatial tasks is the ability to accurately perceive and interpret spatial information from the environment. If the item involves multiple complex shapes, or distinct pieces, or lacks identifiable verbal labels (e.g., “circle”, “triangle”), the EN is coded as “1”. If none of these criteria are met, the EN is coded as “0”. In the OA test, since all fifteen items require encoding ability, all ENs in the first column of the Q-matrix are coded as “1”.

Falsification (FA) involves test takers rejecting or identifying as incorrect those response options that have an incorrect number of parts, incorrect relative sizes of components, or shapes that do not fit together correctly. In the second column (FA) of the Q-matrix, an item is marked with a “1” if these discrepancies exist between the stem and the options.

Mental displacement (MM) and mental rotation (MR) are two core spatial reasoning abilities used to solve OA items. When the position and orientation of objects differ between the stem and options, test takers employ these skills to address the problem. If solving the correct response requires mental movement or mental rotation, the characteristics (MR and MM) of the Q-matrix are marked with a “1”.

For confirmation-intractability (C-I) of the Q-matrix, an item is marked with a “1” if it requires multi-step operations to solve the question, including both mental movement and mental rotation. The expected number of comparison cycles to find a mismatch from the stem to the distractor and the proportion of pieces that are mismatched by small angular disparities.

#### 2.4.2. The Lognormal RT Model

To model the time used for decoding each spatial reasoning item, we selected the lognormal RT model (Van der Linden 2006) for RT analysis in this study. Let $T_{ni}$ be the observed RTs of person n to item i, and the lognormal RT model can be expressed as

(7) $$T_{ni}∼f(t_{ni},\tau_{n},\omega_{i},\xi_{i})=\frac{\omega_{i}}{t_{ni}\sqrt{2\pi }}\exp(-\frac{\omega_{i}^{2}}{2}{\left(\log t_{ni}-(\xi_{i}-\tau_{n})\right)}^{2}),$$

in which $\log t_{ni}$ is the logarithm of RTs; $\tau_{n}$ is the latent processing speed of person n when completing a task and is assumed to be normally distributed with a mean of zero and a variance, $\sigma_{\tau }^{2}$; $\xi_{i}$ is the time-intensity of item *i* or the average time needed across all test-takers to complete item i; $\omega_{i}$ is the reciprocal of the standard deviation of the error term, which is treated as a time-precision parameter. It is important to note that as τ increases, the person tends to allocate less time to each item.

#### 2.4.3. The Negative Binomial Fixation (NBF) Model

NBF model (Man and Harring 2019) was proposed to analyze discrete fixation counts, because it incorporates individualized latent personalized visual engagement parameters, which can be used to reveal each participant’s level of visual engagement on decoding items and simultaneously show item-level characteristics. This approach reflects the extent of visual effort required from respondents to complete an item, along with its discriminating power among them.

(8) $$V_{ni}∼f(v_{ni},\varepsilon_{n},h_{i},m_{i})=\frac{\Gamma (v_{ni}+h_{i})}{v_{ni}!\Gamma (h_{i})}{\left(\frac{h_{i}}{\exp(\varepsilon_{n}+m_{i})+h_{i}}\right)}^{h_{i}}{\left(\frac{\exp(\varepsilon_{n}+m_{i})}{\exp(\varepsilon_{n}+m_{i})+h_{i}}\right)}^{v_{i}},$$

in which $\epsilon_{n}$ is the latent visual engagement of person n, which represents the overall visual engagement level paid to the test; $m_{i}$ is the visual intensity of item i, which represents the averaged amount of visual engagement needed to complete an item; $h_{i}$ is the dispersion or shape parameter of the expectation of the random variable $V_{ni}$, where $E(V_{ni})=\mu_{ni}=\exp(\varepsilon_{n}+m_{i})$. In cases where a given item has a fixed $m_{i}$, a higher level of visual engagement would result in a proportional increase in FCs. Conversely, for a given person with fixed $\epsilon_{n}$, items that require a higher amount of visual intensity would also result in higher FCs. Furthermore, a visual-discrimination parameter for item i is defined as $d_{i}=1/\sqrt{\mu_{i}+\mu_{i}^{2}/h_{i}}$, where $\mu_{i=}\sum_{i=1}^{I}\mu_{ni}/I$. The discrimination parameter reflects the overall dispersion of the FCs on item i: Larger values lead to steeper slopes of the probability mass function of the negative binomial distribution, while smaller values correspond to flatter slopes.

#### 2.4.4. Assumptions

According to Zhan et al. (2018), $\mu_{\delta }$ is constrained to be positive to satisfy the monotonicity requirement. This implies that for more than half of the items, an increased mastery of the required attributes enhances the probability of a correct response.

In the MJ-DINA model, $\mu_{\theta }=\mu_{\tau }=\mu_{\epsilon }=0$ and $\sigma_{\theta }^{2}=1$ are set to ensure that three person parameters are in same scale, and to ensure scale identifiability. Meanwhile, there are no constraints on the mean and variance of the item parameters nor the variances of the other two person parameters (Zhan et al. 2022).

Under joint-hierarchical modeling, eight conditional independence assumptions are made: (1) $\alpha_{nK}$ are independent given $\theta_{n}$, a core concept of the higher-order latent structural model; (2) $Y_{ni}$s are independent given $\alpha_{n}$, fundamental to the DINA model; (3) $log(T_{ni})$s are independent given $\tau_{n}$, key to the lognormal RT model; (4) $V_{ni}$s are independent given $\epsilon_{n}$, central to the NBF model; (5–8) Various assumptions ensure observed variables are influenced solely by specific person parameters, and indicate a simple-structure hierarchical modeling without cross-loading. This framework uses distinct measurement models for RA, RT, and FC, each fully explained by their respective latent variables.

The MJ-DINA Model, by considering accuracy, response times and fixation counts, provides a thorough examination of spatial abilities. When we assess these aspects together it aids in comprehending and discovering the most suitable problem-solving strategies to assist individuals in achieving improved scores. Moreover, this model could enhance the prediction of a person’s spatial ability better by combining different supporting information sources. The MJ-DINA Model, with its diverse measurements, is a powerful tool for educators and researchers aiming to enhance and understand spatial reasoning capacities.

#### 2.4.5. Bayesian Estimation

We implemented Markov chain Monte Carlo (MCMC) estimation for the MJ-DINA model parameters within a Bayesian framework. The distribution of priors varies across three sub-models: the DINA model, the lognormal RT model, and the NBF model. The validity of the MJ-DINA model has been established in previous studies (Man and Harring 2019; Zhan et al. 2022). We applied the prior settings in accordance with the previous results. Consequently, $Y_{ni}$, $log\left(T_{ni}\right)$, $V_{ni}$, and $\alpha_{nk}$ are assumed to be distributed as follows:

(9) $$Y_{ni}∼Bernoulli(P(Y_{ni}=1)),$$

(10) $$\log(T_{ni})\pi ∼N(\xi_{i}-\tau_{n},\omega_{i}^{-2}),$$

(11) $$V_{ni}∼NB(\exp(\varepsilon_{n}+m_{i}),d_{i}^{-2}),$$

(12) $$\alpha_{nk}∼Bernoulli(P(\alpha_{nk}=1)).$$

The priors of the four item parameters are assumed from a multivariate normal distribution as

(13) $$\left(\begin{array}{c} \beta_{i}\\ \delta_{i}\\ \xi_{i}\\ m_{i} \end{array}\right)∼\left(\left(\begin{array}{c} \mu_{\beta }\\ \mu_{\delta }\\ \mu_{\xi }\\ \mu_{m} \end{array}\right),\sum_{item}\right).$$

The priors selected for modeling were chosen based on their appropriateness and relevance. We reviewed and assessed various potential priors from existing research in cognitive assessment, with a particular focus on spatial reasoning tests. According to Zhan et al. (2018), and Man and Harring (2019), we set intercept and interaction in the DINA model as values of −1.096 and 2.219 following a normal distribution, respectively. We used the average logarithm of response time and fixation counts from our data, which were 3 (equal to about 20 s for answering an item) and 2 (equal to about 12 fixation counts for decoding an item). This was done because response time and visual engagement varied between tests (Man et al. 2022). The hyperpriors are specified as follows:

(14) $$\mu_{\beta }∼N(-1.096,2),$$

(15) $$\mu_{\delta }∼N(2.219,1)I(\mu_{\delta }>0),$$

(16) $$\mu_{\xi }∼N(3,1),$$

(17) $$\mu_{m}∼N(2,1),$$

(18) $$\sum_{item}∼InvWishart(R,4),$$

in which, $\mu_{\beta }$ and $\mu_{\delta }$ are intercept and interaction in the DINA model; $\mu_{\xi }$ is the mean logarithm response time in the lognormal RT model; $\mu_{m}$ is the averaged log-transferred fixation counts, reflecting different levels of visual engagement, in the NBF model; R is defined as a four-dimensional identity matrix, represented as a 4 × 4 matrix with 1 s on its main diagonal and 0 s elsewhere. For the other two item parameters, the priors are established as $\omega_{i}^{-2}\sim InvGamma(1,1)$ and $h_{i}\sim InvGamma(1,1)$.

In addition, the priors of three person parameters are set as

(19) $$\left(\begin{array}{c} \theta_{n}\\ \tau_{n}\\ \varepsilon_{n} \end{array}\right)∼MVN\left(\left(\begin{array}{c} \theta_{n}\\ \tau_{n}\\ \varepsilon_{n} \end{array}\right),\Sigma_{person}\right),$$

(20) $$\Sigma_{person}=\Delta_{person}{\Delta^{\prime }}_{person},$$

(21) $$\left(\begin{array}{c} \theta_{n}\\ \tau_{n}\\ \varepsilon_{n} \end{array}\right)=\left(\begin{array}{ccc} 1 & & \\ \varphi_{21} & \varphi_{2} & \\ \varphi_{31} & \varphi_{32} & \varphi_{3} \end{array}\right),$$

in which, the Cholesky decomposition of the $\Sigma_{person}$ is used because the variance of $\theta_{n}$, is set to 1 for identification (Zhan et al. 2018). ${∆}_{person}$ is a low triangular matrix with positive entries on the diagonal and unrestricted entries below the diagonal, and ${∆\prime }_{person}$ is the conjugate transposition of ${∆}_{person}$. The priors for ${∆}_{person}$ are specified as $\varphi_{21}\sim N(0,1)$, $\varphi_{31}\sim N(0,1)$, $\varphi_{32}\sim N(0,1)$, $\psi_{2}\sim Gamma(1,1)$, and $\psi_{3}\sim Gamma(1,1)$. Furthermore, the priors for the higher-order latent structure parameters are set as $\lambda_{k}\sim N(0,4)$ and $\gamma_{k}\sim N(0,4)I(\gamma_{k}>0)$.

#### 2.4.6. Coding

The primary coding environment used was R 4.3.3 (Venables and Smith 2003), a widely recognized programming language in the fields of statistics and educational research (Ihaka and Gentleman 1996). Due to the small sample size and complex model structure, MJ-DINA model (Zhan et al. 2019) was estimated using the Just Another Gibbs Sampler software (JAGS 4.3.1; Plummer 2023) through the rjags package (Plummer 2017) in R. After estimating parameters of MJ-DINA, we employed the mcmcplots package (Curtis et al. 2015) for visualization and coda package (Plummer et al. 2006) for MCMC diagnostics and sample generation in R 4.3.3 (Venables and Smith 2003).

## 3. Results

### 3.1. Data Description and Analysis

**Figure 3:**
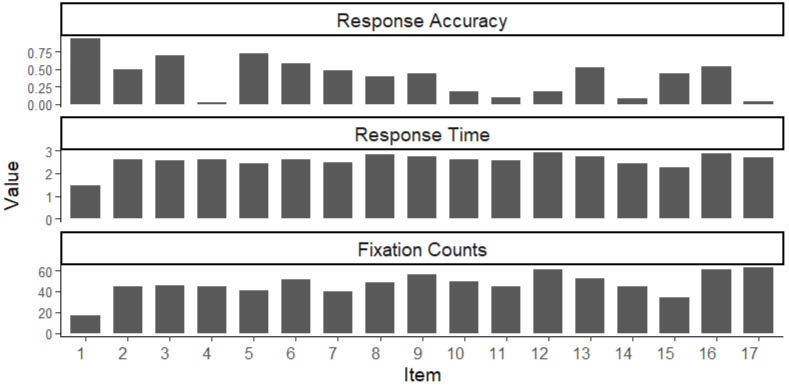
Descriptive Statistics Summary for Response Accuracy, Response Time, and Fixation Counts.

In the top panel of Figure 3, Classical Test Theory (CTT) was used to analyze response accuracy (RA) for all seventeen items. The items ranged in accuracy rate from 94% for item 1 to 2% and 4% for item 4/17, suggesting a reasonable range of accuracy rates across items, reflecting varying levels of difficulty.

In the middle panel, item 1 exhibited lower logarithm response time (LogT), suggesting that participants spent less time completing this trial (*M* = 1.46). Meanwhile, items 12 and 16 required longer average response times (*M* = 2.90 and 2.86, respectively), implying that participants generally spent more time on these items.

Regarding fixation counts (FCs), the results indicate item 17 had the highest mean fixation count (*M* = 62.92), which means these items require more intensive visual exploration or involve more complex stimuli. Interestingly, RA, LogT, and FCs do not show strong correlations. The relationship between the three variables may vary across different items, suggesting that these statistics provide distinct information about examinees’ cognitive processes. This prompted us to further examine the results of the MJ-DINA model.

### 3.2. Result of MJ-DINA Model

The MJ-DINA model parameters were estimated using MCMC sampling, utilizing three parallel chains with 100,000 iterations each. After discarding the first 50,000 iterations as burn-in, the remaining 50,000 iterations per chain were used for posterior inference. To reduce autocorrelation, we applied a thin interval of 10 using the coda package (Plummer et al. 2006).

#### 3.2.1. Convergence Diagnostics and Model Fit

Modeling convergence was assessed using the Gelman–Rubin statistic, also known as $\hat{R}$. As shown in Table 4, all item parameters demonstrated $\hat{R}$ values approximately equal to 1.00, indicating adequate convergence to the stationary distribution. Typically, $\hat{R}$ below 1.1 or 1.2 indicates good convergence, demonstrating sufficient exploration of the parameter space (Brooks and Gelman 1998).

**Figure 4:**
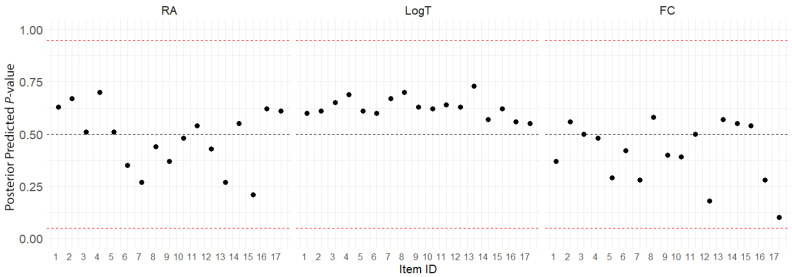
Posterior predictive *p*-values for DINA model (RA), Log RT model (LogT), and negative binomial visual fixation counts model (FCs) over 17 items. The dots represent the statistic-based item-level posterior predictive probability (PPP) values.

The PPMC (Gelman et al. 1996) was utilized to evaluate the absolute model-data fit for the MJ-DINA model. The PPP value, a metric derived from PPMC, represents the probability that the replicated data could exceed the observed data according to the model. Indicator-based and statistic-based PPP are two prevalent approaches to compare observed response with replicated response. Compared to indicator-based PPP, which compares observed response with replicated response directly, statistic-based PPP quantifies the discrepancy between observed and predicted data using test statistics, offering a more precise model fit assessment by accounting for variance. In Figure 4, the three dashed horizontal lines in each plot denote 0.05, 0.5, and 0.95, respectively. All *PPP* values for items across the DINA, Log RT, and NBF models are located between 0.05 and 0.95, indicating that there are no systematic differences between observed and replicated values.

#### 3.2.2. Relationship Between MJ-DINA Parameters

To understand the overall relationship between item parameters, the variance and covariance of the item parameters with credible intervals (CIs) were estimated during the MCMC process (see left panel of Table 5). In the higher-order DINA model, the covariance between the intercept and interaction parameter (i.e., guessing and slipping probability), denoted as $\sigma_{\beta ,\delta }$, was estimated to be 1.582. This positive covariance indicates that as the guessing probability increase, the slipping probability tends to decrease, because slipping parameter is negatively related to δ parameter, indicating an inverse relationship between guessing and slipping. Additionally, $\sigma_{\beta ,m}$ and $\sigma_{\beta ,\xi }$ were estimated to be −0.207 and −0.209, which means that items with higher intercepts (i.e., guessing probability) tended to both involve less time and visual intensity. Interestingly, $\sigma_{\xi ,m}$ was estimated to be 0.087, a comparatively low covariance, which means that items with higher time intensity did not necessarily require higher visual intensity.

The variance and covariance of the person parameters (Table 5) revealed several relationships. The negative covariance between latent ability and visual engagement ($\sigma_{\theta ,\tau }$ = −0.532) indicated that the high spatial ability group demonstrated greater visual engagement through increased eye gaze frequency. Additionally, the positive covariance between latent ability and problem-solving speed ($\sigma_{\theta ,\omega }=$ 0.467) suggested that individuals with high spatial ability tended to spend more time solving OA tasks. Moreover, the covariance between speed and visual engagement showed a mild negative relationship ($\sigma_{\tau ,\omega }$ = −0.195), indicating that participants who spent more time on tasks tended to exhibit fewer eye movements.

**Figure 5:**
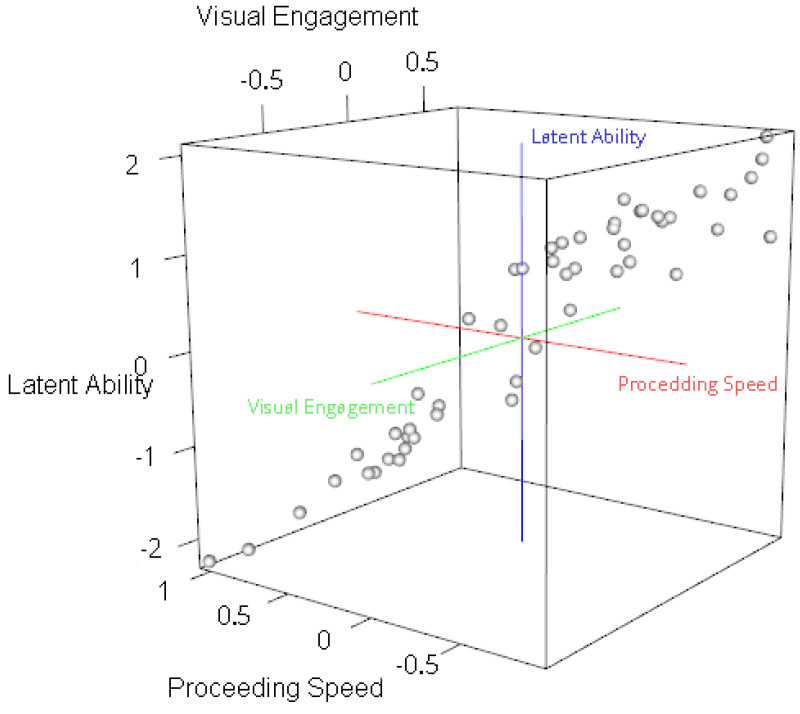
Scatter Plot with Latent Ability, Processing Speed, and Visual Engagement. Proceeding Speed (τ) is the x-axis; Visual Engagement (ε) is the y-axis; Latent Ability (θ) is the z-axis.

This relationship is further illustrated in Figure 5. Latent spatial ability tends to increase as you move from the lower left corner of the plot (representing faster processing speed and lower visual engagement) towards the upper right corner (representing slower processing speed and higher visual engagement). In other words, individuals who solve spatial tasks more slowly and exhibit greater visual engagement tend to show an increase in latent ability.

#### 3.2.3. Item Parameter Estimation

Zhan et al. (2022) addressed that in the DINA model, the intercept parameter (β) and interaction parameter (δ) influence the slipping and guessing probabilities. A positive intercept (β) indicates a higher guessing probability ($g_{i}>0.5$), suggesting that item characteristics or external cues may allow correct responses without full mastery of the required skills. Meanwhile, a non-negative interaction (δ) supports the monotonicity assumption, ensuring that increased mastery reduces slipping by maintaining consistent item difficulty. In Table 6, for the higher-order DINA model, the item intercept parameter (β) for the first item is obviously higher than others ($\beta_{1}$ = 1.82), which means that the guessing probability of this item was the highest ($g_{1}$ = 0.85). A high guessing parameter suggests that a correct response to these items may depend on additional mental resources or unmeasured attributes (Maris 1999). The interaction parameter (δ) for items 4, 11, 14, and 17 is negative, causing extremely high slipping parameters ($s_{4}$ = 0.97, $s_{11}=$ 0.89, $s_{14}=$ 0.92, $s_{17}$ = 0.95), which means some examinees who master all attributes may probably miss the correct answer.

Additionally, the time-intensity parameter (ξ) and visual-intensity parameter (*m*) showed similar patterns across items. Item 1 demonstrated the lowest time-intensity parameter ($\xi_{1}$ = 1.46), while other items clustered around 2.60 with low standard deviations, ranging from 0.10 to 0.13. Similarly, Item 1 exhibited the lowest visual-intensity parameter ($m_{1}$ = 2.83) while other items averaged approximately 3.70 with low standard deviations, ranging from 0.09 to 0.12.

### 3.3. Inferring Spatial Strategies from DINA Estimates

To better understand how individuals with varying levels of latent spatial ability choose problem-solving strategies, we sought to categorize them into distinct groups to analyze their behavioral patterns and develop tailored spatial development education. While several existing eye-tracking-based problem-solving strategy theories have been explored in educational contexts, we select a reliable and effective framework, Kagan and Gardner’s classification, which has been proved by Zhan et al. (2022) and Man et al. (2022). According to Kagan’s (1965) classification, respondents were divided into two cognitive styles: reflective, characterized by taking more time to solve problems, and impulsive, characterized by using less time. In addition, Gardner et al. (1959) categorized respondents as scanners, who have high fixation counts, and focusers, who have low fixation counts. Based on persons’ proceeding speed parameter (τ) and visual engagement parameter (ε), individuals can be divided into four categories: Impulsive-Focuser (I-F), Impulsive-Scanner (I-S), Reflective-Focuser (R-F), and Reflective-Scanner (R-S).

In our study, as shown in Table 7, most participants (*N* = 26) belonged to the Reflective-Scanner (R-S) group, which has the highest spatial ability (θ = 1.11), the slowest processing speed (τ = −0.41) and the most intense visual engagement (ε = 0.37). Meanwhile, the Impulsive-Focuser (I-F) group with 19 children was the second biggest group, which has the lowest spatial ability (θ = −1.09) with the fastest response speed (τ = 0.46) and the least interest in moving their gaze (ε = −0.41).

**Figure 6:**
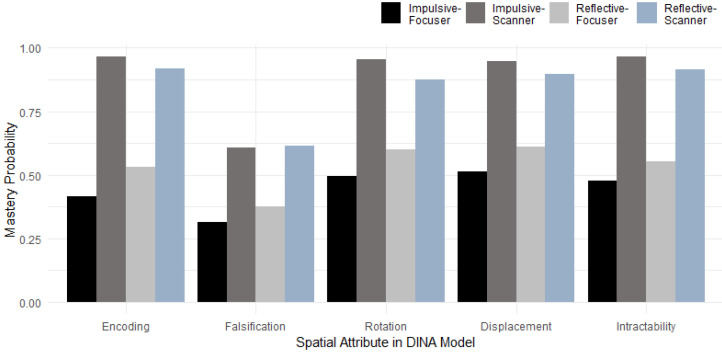
Mastery Probability of Attributes across Problem-Solving Strategy Categories.

More than higher-order latent spatial ability, processing speed, and visual engagement, the MJ-DINA model also provided an analysis of specific spatial reasoning attributes. Figure 6 illustrates that individuals employing different problem-solving strategies exhibit varying probabilities of mastering these spatial reasoning attributes. Notably, the Impulsive-Focuser (I-F) group, which was estimated to have the lowest higher-order spatial ability, unsurprisingly showed the lowest probability of mastering spatial attributes, particularly in encoding. Interestingly, the Reflective-Scanner (R-S) group, despite being estimated to have the highest latent spatial ability, did not exhibit the highest probability of mastering all spatial attributes. Instead, the Impulsive-Scanner (I-S) group consistently demonstrated the highest mastery probability for four spatial attributes, except falsification. A possible explanation for this is that, although individuals in the I-S group have the highest probability of mastering most spatial attributes, the fast processing speed may negatively impact their performance on the OA test. Another potential explanation is that falsification is a critical factor in improving individual performance in spatial tasks. Of course, we need to pay attention to the small sample size for the I-S group (*N* = 2, see Table 7), which may not be sufficient to prove this result.

Additionally, the probability of mastering the falsification attribute was the lowest compared to other attributes across all four problem-solving strategy groups. This suggests that there is greater potential for improvement in mastering the falsification attribute, which could help students enhance their performance on spatial reasoning tasks.

**Figure 7:**
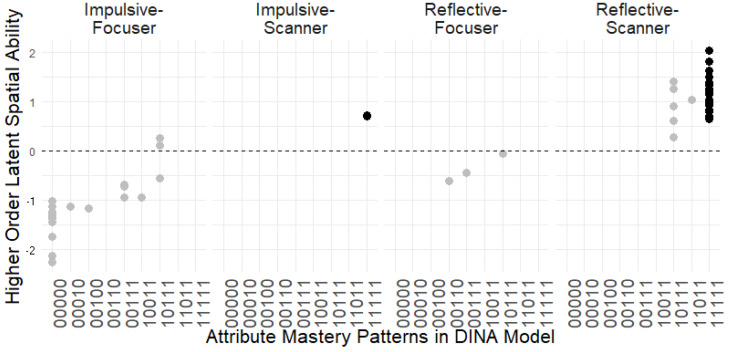
Spatial Reasoning Ability Across Different Strategies and Attributes Mastery Patterns. Higher-order latent spatial ability is plotted on the y-axis, with dashed line at *y* = 0 indicating the median for latent ability. Attribute mastery patterns in the DINA model is on the x-axis. Four columns are problem-solving strategies: Impulsive-Focuser, Impulsive-Scanner, Reflective-Focuser, and Reflective-Scanner. The pattern “11111” is represented by black dots, while all other patterns are shown as grey dots.

The results identified nine spatial reasoning attribute patterns: “00000”, “00010”, “00100”, “00110”, “00111”, “10011”, “10111”, “11011”, and “11111”. Figure 7 illustrates that Reflective-Scanners generally outperform Impulsive-Focusers, indicating that increased response time and fixation counts are associated with better performance on spatial reasoning tasks.

Individuals who mastered all spatial attributes (“11111”) were more likely to adopt a Reflective-Scan strategy, characterized by extended response times and increased eye movement. Notably, all individuals classified as Impulsive-Focusers did not master the falsification attribute, as indicated by the “0” in the second position of their mastery patterns. Therefore, the lack of falsification mastery could be a key factor contributing to their shorter response times and higher fixation counts.

Our finding revealed that the two Impulsive-Scanners were estimated to have “11111” pattern with high latent spatial ability, indicating they spent less time but sufficient eye movements to perform well on OA tasks. However, due to the small sample size, we cannot conclusively determine whether the Impulsive-Scanner strategy, characterized by rapid eye movements, is a more efficient method for solving OA tasks. Nevertheless, we can conclude that increased gaze movement is a strong indicator of a higher probability of mastering more spatial reasoning attributes and achieving better performance on OA tests, as it suggests greater visual engagement in processing spatial information. Furthermore, frequent gaze movement was a stronger predictor of success in OA tests than response speed.

## 4. Discussion

Our research employed the MJ-DINA model (Man and Harring 2019; Zhan et al. 2022) to enhance spatial ability assessment, accessing a comprehensive understanding of individuals’ performance in spatial tasks. Compared to traditional cognitive diagnosis modeling, our approach offers information about individuals’ intentions for problem-solving strategies, which can assist educators and trainers in designing and implementing appropriate methods to improve students’ spatial abilities. In the following sections, we will discuss our results as they relate to our predetermined research questions. Then, we discuss the implications and limitations of research and practice, as well as some potentially meaningful future research directions.

### 4.1. Research Question 1

Our findings demonstrate the effectiveness of the MJ-DINA model in assessing spatial ability, supporting prior work showing that accuracy-only measures fail to capture problem-solving strategies (Shepard and Metzler 1971; Nazareth et al. 2019). The model fits the data well, showing adequate model convergence and item fit. Importantly, the item and person parameters estimated under the MJ-DINA model were not highly correlated, suggesting that they provide unique information about the items and respondents.

The Gelman–Rubin statistic values of approximately 1.00 for all item parameters indicated adequate convergence of the MCMC chains and reliable model parameter estimation. Continually, the PPMC results showed that all posterior predictive *p*-values for the DINA, Log RT, and NBF models fell within the acceptable range of 0.05 to 0.95, indicating the adequacy of the model’s fit to the data. These findings suggest that the Bayesian estimation method can effectively recover the parameters of the MJ-DINA model even with a limited sample size, resulting in a good fit to the observed data on spatial reasoning performance, response times, and fixation counts.

### 4.2. Research Question 2

We classified all participants into four different problem-solving strategy categories based on response time and fixation count: Impulsive-Focuser, Impulsive-Scanner, Reflective-Focuser, and Reflective-Scanner. Additionally, we found that most high-performing participants fell into the Reflective-Scanner category while low-performing participants were typically Impulsive-Focusers. This suggests that a reflective, careful approach enhances spatial task performance. This finding aligns with Nazareth et al.’s (2019) results, that the piecemeal strategy (i.e., switching eye-pattern, scanner) leads to better performance than the holistic strategy (i.e., fixating eye-pattern, focuser) in spatial reasoning tasks.

Interestingly, the Impulsive-Scanner group (*N* = 2), though only two participants, demonstrated the ability to achieve high scores in a shorter time, which is intriguing and suggests potential support for a subgroup of students using a “leaping” strategy. This may involve using heuristics (Mitchell et al. 1983; Salhi 2017) to quickly eliminate incorrect options or employing Gestalt perception (Jäkel et al. 2016; Wagemans et al. 2012) to process spatial information holistically. This complexity underscores the need for a higher-order DINA model that captures individual differences in cognitive processes.

### 4.3. Research Question 3

To answer the third question, we utilized a higher-order DINA model in the MJ-DINA model to explore the impact of general problem-solving strategies on individuals’ performance in spatial attributes (e.g., encoding). Our results indicate that the falsification attribute had the lowest mastery rate across all four problem-solving strategy groups, yet it was the strongest predictor of spatial reasoning success. This result aligns with previous research showing that effective problem solvers spend more time to check errors and test hypotheses, a crucial aspect of falsification (Popper 1963; Lam 2007). Moreover, individuals who tend to use falsification outperform those who solely rely on confirmation bias (Nickerson 1998). Our findings extend this idea into the spatial reasoning domain, demonstrating that falsification may be the key differentiator in high spatial reasoning performance. However, in the previous theory that mental rotation is the critical determinant of spatial ability (Shepard and Metzler 1971), our findings suggest that falsification is more critical. In future studies, we should investigate whether explicit training in falsification strategies can improve spatial task performance, especially for OA tasks.

## 5. Conclusions

### 5.1. Implications

The MJ-DINA model, integrating response time and eye-tracking data, can significantly support educational applications, particularly in STEM education. Educators and psychologists may be able to more deeply understand how students’ spatial abilities interact with their problem-solving approach and design personalized interventions to enhance their spatial reasoning skills. The first implication is that educators, psychologists, and curriculum designers could use tailored teaching and training methods to improve the specific spatial abilities (e.g., coding, falsification, mental rotation, displacement, and intractability) of individuals based on the results of the DINA model. For example, Students struggling with mental rotation could be supported with progressive rotation exercises that gradually increase in complexity (e.g., starting with 90° rotations before advancing to 3D rotations). The second important implication is that the MJ-DINA model may help to identify specific attributes of spatial ability where interventions are required to enhance a person’s strategies for solving spatial problems in STEM education. For instance, students classified as Impulsive-Focusers might benefit from explicit training in falsification skills, such as structured comparison exercises where they must systematically eliminate incorrect spatial configurations. Some students relying on intuitive scanning may need strategy refinement exercises that encourage systematic scanning patterns rather than rapid heuristic-based elimination.

Additionally, based on four problem-solving strategies, educators and trainers potentially offer personalized training for individuals within each group. For the Reflective Scanners group, we can impose time constraints on spatial reasoning tasks to enhance efficiency and evaluate accuracy discrimination. While Reflective-Focusers behaviors could be caused by a few things, such as not having the necessary spatial skills, getting confused about the stimuli, or lacking engagement, we could use interactive, dynamic, and different spatial tasks to help them understand tasks easily and engage. For Impulsive-Scanners, we might introduce organized comparison exercises that require them to pause and review their choices before submitting the response. For Impulsive-Focusers, we could use guided eye-tracking feedback that highlights which areas they should focus on during tasks.

### 5.2. Limitations

This study has certain restrictions. First, our study includes only 50 participants aged 6–14, which limits the generalizability of the findings to broader populations. This is particularly evident for certain problem-solving strategy groups, such as Impulsive-Scanner (*N* = 2) and Reflective-Focuser (*N* = 3). Moreover, the narrow age range of participants restricts generalizability. Future research should consider incorporating a larger sample size and including younger and adult participants to explore more variation in the developmental stage. Additionally, cultural and educational backgrounds are critical factors in spatial reasoning and problem-solving strategy selection, yet we did not explicitly control them in our study. Previous research suggests that educational experience, curriculum emphasis on spatial reasoning, and cultural environment significantly influence spatial ability development (Uttal et al. 2013). Future studies should include participants from diverse educational systems and cultural contexts to examine whether strategy preferences vary across different populations.

Although the MJ-DINA model provided a multi-faceted analysis of participants’ cognitive processes in spatial reasoning tasks, it may not capture all latent factors affecting OA task performance out of the Q-matrix design. In addition, in terms of problem-solving strategies, this study focused only on response time and fixation counts, not including other eye-tracking metrics and potential behavioral patterns that could offer a more comprehensive understanding of strategy selection. Future research could incorporate additional eye-tracking data to enrich the analysis of problem-solving strategies.

### 5.3. Future Research

Firstly, to validate the MJ-DINA model in assessing spatial abilities and to investigate the variations in spatial skills and problem-solving techniques among diverse groups, we need to broaden our sample size and diversity, incorporating various ages, educational backgrounds, and cultural contexts. Moreover, individuals usually make strategic shifts when they seek a balance between accuracy and speed, which may affect how well they complete the task (Ackerman and Kanfer 2009; Kyllonen and Christal 1990). We could look at strategy changes to learn more about how people modify their strategies to solve problems in response to different task demands and time restrictions.

Moreover, our results demonstrate that certain items exhibited significantly higher guessing parameters, suggesting that a correct answer may be affected by extra latent cognitive abilities or omitted attributes beyond the designated Q-matrix (Maris 1999). Incorporating more valid factors into the MJ-DINA model would improve the model’s predictive validity and provide more instructional recommendations for spatial education. For example, working memory is a key fluence for individuals to deal with complex spatial problems (Miyake and Shah 1999), yet emotional control plays a crucial role in facing pressure, particularly for time-sensitive activities (Petrova and Gross 2023).

Finally, eye-tracking techniques provided not only fixation counts but also an array of supplementary data, such as fixation duration, visit duration, visit count, and gaze patterns, all of which have been demonstrated to enhance the analysis of problem-solving strategies (Nazareth et al. 2019; Holmqvist et al. 2011). In future studies, we could include metrics like fixation time and gaze patterns, to explore more complex cognitive processes and strategy shifts during solving spatial problems.

## Figures and Tables

**Table 1:** Overview of Spatial Cognitive Processes.

Author	Spatial Cognitive Processes			
Cooper and Shepard (1973)	Encoding	Mental Rotation	Confirmation	Motor Response
Pellegrino et al. (1985)	Falsification	Mental Rotation	Verification	
Embretson and Gorin (2001)	Encoding	Falsification	Confirmation	

**Table 2:** Proposed Cognitive Processes in Spatial Tasks.

No.	Stage	Description
A1	Encoding	More than three pieces, curved edges, and standard shapes (e.g., circle, triangle).
A2	Falsification	Distractors with obvious mismatches (e.g., wrong number of pieces, sizes, shapes).
A3	Confirmation-Rotation	The confirmation stage for rotation depends on the number of rotated pieces.
A4	Confirmation-Displacement	At least two pieces must be substantially moved into place for the correct answer
A5	Confirmation-Intractability	Distractors only ruled out by subtle mismatches, requiring attention to small angular disparities.

**Table 3:** The Q-matrix of Spatial Ability of OA Test.

Item	EN	FA	C-R	C-D	C-I
1	1	0	1	0	1
2	1	0	1	1	1
3	1	0	1	1	1
4	0	0	1	0	1
5	0	1	1	0	1
6	1	1	1	1	1
7	0	0	1	1	1
8	1	0	1	0	1
9	1	0	0	0	1
10	0	0	1	1	1
11	1	1	1	0	1
12	1	1	1	1	1
13	1	1	1	0	1
14	1	0	1	0	1
15	1	1	1	1	1
16	1	0	0	1	1
17	1	0	0	1	1

Note. EN = Encoding; FA = Falsification; C-R = Confirmation-Rotation; C-D = Confirmation-Displacement; C-I = Confirmation-Intractability. A “1” indicates that the item is required for this ability, while a “0” signifies that this ability is not needed for the item.

**Table 4:** Gelman–Rubin statistic $\left(\hat{R}\right)$ for Item Parameters in the MJ-DINA Model.

Item	β	δ	g	s	ξ	m
1	1.00	1.08	1.00	1.01	1.01	1.01
2	1.02	1.00	1.01	1.01	1.01	1.01
3	1.02	1.02	1.02	1.01	1.01	1.01
4	1.02	1.08	1.02	1.05	1.01	1.01
5	1.02	1.09	1.03	1.01	1.01	1.01
6	1.03	1.05	1.03	1.01	1.01	1.01
7	1.02	1.02	1.02	1.00	1.01	1.01
8	1.03	1.01	1.03	1.00	1.01	1.01
9	1.01	1.01	1.01	1.00	1.01	1.01
10	1.06	1.04	1.03	1.00	1.01	1.01
11	1.03	1.17	1.03	1.03	1.01	1.01
12	1.03	1.02	1.03	1.00	1.01	1.01
13	1.04	1.04	1.04	1.03	1.01	1.01
14	1.04	1.06	1.02	1.01	1.00	1.01
15	1.02	1.02	1.01	1.02	1.01	1.01
16	1.02	1.01	1.01	1.01	1.00	1.01
17	1.04	1.09	1.03	1.02	1.00	1.00

Note. β = intercept in DINA (RA) model; δ = interaction in DINA (RA) model; g = guessing in DINA (RA); s = slipping in DINA (RA); ξ = time-intensity in Log RT model; m = visual-intensity in NBF (FC) model.

**Table 5:** Variance–Covariance of Posterior Distribution Estimates for Item and Person Parameters.

Item	Variance–Covariance Parameter		Person	Variance–Covariance Parameter	
	Mean	CI		Mean	CI
$\sigma_{\beta }^{2}$	1.699	(0.482, 3.313)	$\sigma_{\theta }^{2}$	2.387	(1.004, 4.498)
$\sigma_{\delta }^{2}$	2.983	(0.143, 9.759)	$\sigma_{\tau }^{2}$	0.253	(0.148, 0.365)
$\sigma_{\xi }^{2}$	0.181	(0.072, 0.318)	$\sigma_{\omega }^{2}$	0.194	(0.114, 0.282)
$\sigma_{m}^{2}$	0.145	(0.060, 0.261)	$\sigma_{\theta ,\tau }$	−0.532	(−1.030, −0.116)
$\sigma_{\beta ,\delta }$	1.582	(−0.001, 4.001)	$\sigma_{\theta ,\omega }$	0.467	(0.102, 0.896)
$\sigma_{\beta ,\xi }$	−0.243	(−0.607, 0.036)	$\sigma_{\tau ,\omega }$	−0.195	(−0.288, −0.115)
$\sigma_{\beta ,m}$	−0.207	(−0.518, 0.056)			
$\sigma_{\delta ,\xi }$	−0.209	(−0.773, 0.191)			
$\sigma_{\delta ,m}$	−0.184	(−0648, 0.186)			
$\sigma_{\xi ,m}$	0.087	(0.005, 0.192)			

Note. Mean, mean value of the posterior distribution; CI, credible interval; β, intercept of RDINA; δ, interaction of RDINA; ξ, item-side time intensity; m, item-side visual intensity. θ, higher-order latent ability; τ, speediness; ω, visual engagement.

**Table 6:** Item Parameters Estimates in MJ-DINA Model.

Model	DINA (RA)				Log RT	NBF (FC)
Item	β	δ	g	s	ξ	m
1	1.82 (0.57)	3.77 (1.61)	0.85 (0.07)	0.01 (0.02)	1.46 (0.12)	2.83 (0.10)
2	−0.77 (0.38)	1.94 (0.59)	0.32 (0.08)	0.25 (0.10)	2.55 (0.11)	3.63 (0.09)
3	0.02 (0.38)	2.29 (0.75)	0.51 (0.09)	0.11 (0.06)	2.52 (0.11)	3.71 (0.09)
4	−2.92 (0.70)	−1.16 (1.13)	0.06 (0.04)	0.97 (0.03)	2.58 (0.10)	3.69 (0.10)
5	0.34 (0.44)	2.55 (1.15)	0.58 (0.10)	0.09 (0.07)	2.38 (0.11)	3.60 (0.10)
6	−0.16 (0.37)	1.85 (0.94)	0.46 (0.09)	0.20 (0.12)	2.59 (0.11)	3.75 (0.10)
7	−0.70 (0.37)	1.11 (0.55)	0.34 (0.08)	0.40 (0.11)	2.44 (0.10)	3.56 (0.09)
8	−1.07 (0.35)	1.23 (0.49)	0.26 (0.07)	0.46 (0.10)	2.77 (0.10)	3.82 (0.09)
9	−0.97 (0.37)	1.17 (0.49)	0.28 (0.07)	0.45 (0.09)	2.71 (0.11)	3.87 (0.10)
10	−1.68 (0.44)	0.30 (0.61)	0.17 (0.05)	0.79 (0.08)	2.58 (0.11)	3.76 (0.10)
11	−2.11 (0.44)	−0.39 (1.13)	0.11 (0.04)	0.89 (0.08)	2.52 (0.11)	3.70 (0.09)
12	−1.64 (0.36)	0.29 (0.70)	0.17 (0.05)	0.77 (0.12)	2.85 (0.11)	3.96 (0.10)
13	−0.41 (0.37)	1.67 (0.93)	0.40 (0.09)	0.26 (0.15)	2.71 (0.10)	3.85 (0.08)
14	−2.18 (0.51)	−0.57 (0.92)	0.11 (0.04)	0.92 (0.05)	2.41 (0.12)	3.71 (0.11)
15	−0.56 (0.33)	0.86 (0.76)	0.37 (0.07)	0.44 (0.16)	2.24 (0.11)	3.43 (0.10)
16	−0.76 (0.39)	1.94 (0.60)	0.32 (0.08)	0.25 (0.10)	2.80 (0.12)	3.95 (0.11)
17	−2.66 (0.57)	−0.79 (1.04)	0.07 (0.04)	0.95 (0.04)	2.69 (0.13)	3.88 (0.12)

Note. Standard error (standard deviation of the posterior distribution) is in parenthesis. In DINA (RA) model, β = intercept; δ = interaction; g = guessing; s = slipping. In Log-RT (RT) model, ξ = time intensity. In NBF (FC) model, m = visual intensity.

**Table 7:** Classification of Problem-Solving Categories Based on MJ-DINA Model.

Category	N	θ	τ	ε
Impulsive-Focuser (I-F)	19	−1.09 (0.63)	0.46 (0.23)	−0.41 (0.22)
Impulsive-Scanner (I-S)	2	0.72 (0.01)	0.03 (0.03)	0.02 (0.00)
Reflective-Focuser (R-F)	3	−0.37 (0.28)	−0.04 (0.06)	−0.05 (0.03)
Reflective-Scanner (R-S)	26	1.11 (0.39)	−0.41 (0.24)	0.37 (0.21)

Note. Standard error (standard deviation of the posterior distribution) is in parenthesis. N is the number of examinees. In MJ-DINA (RA) model, θ = latent ability; τ = processing speed; ε = visual engagement.

## Data Availability

Available on request through the third author.

## References

[B1-jintelligence-13-00030] Ackerman Phillip L., Kanfer Ruth (2009). Test length and cognitive fatigue: An empirical examination of effects on performance and test-taker reactions. Journal of Experimental Psychology: Applied.

[B2-jintelligence-13-00030] Anderson Charles H., Essen David C. Van, Olshausen Bruno A. (2005). Directed visual attention and the dynamic control of information flow. Neurobiology of Attention.

[B3-jintelligence-13-00030] Bornstein Robert F. (2011). Toward a process-focused model of test score validity: Improving psychological assessment in science and practice. Psychological Assessment.

[B4-jintelligence-13-00030] Brooks Stephen P., Gelman Andrew (1998). General methods for monitoring convergence of iterative simulations. Journal of Computational and Graphical Statistics.

[B5-jintelligence-13-00030] Carroll John Bissell (1993). Human Cognitive Abilities: A Survey of Factor-Analytic Studies.

[B6-jintelligence-13-00030] Cooper Lynn A., Shepard Roger N. (1973). Chronometric studies of the rotation of mental images. Visual Information Processing.

[B7-jintelligence-13-00030] Curtis S. McKay, Goldin Ilya, Evangelou Evangelos (2015). mcmcplots: Create Plots from MCMC Output.

[B8-jintelligence-13-00030] De La Torre Jimmy (2011). The Generalized DINA Model Framework. Psychometrika.

[B9-jintelligence-13-00030] De La Torre Jimmy, Douglas Jeffrey A. (2004). Higher-order latent trait models for cognitive diagnosis. Psychometrika.

[B10-jintelligence-13-00030] Embretson Susan, Gorin Joanna (2001). Improving construct validity with cognitive psychology principles. Journal of Educational Measurement.

[B11-jintelligence-13-00030] Gardner Riley W., Holzman Philip S., Klein George S., Linton Harriet P., Spence Donald P. (1959). Cognitive control: A study of individual consistencies in cognitive behavior. Psychological Issues.

[B12-jintelligence-13-00030] Gelman Andrew, Meng Xiao-Li, Stern Hal (1996). Posterior predictive assessment of model fitness via realized discrepancies. Statistica Sinica.

[B13-jintelligence-13-00030] Gorin Joanna S. (2006). Test design with cognition in mind. Educational Measurement: Issues and Practice.

[B14-jintelligence-13-00030] Haertel Edward H. (1989). Using restricted latent class models to map the skill structure of achievement items. Journal of Educational Measurement.

[B15-jintelligence-13-00030] Halpern Diane F. (2000). Sex Differences in Cognitive Abilities.

[B16-jintelligence-13-00030] Hawes Zachary, Ansari Daniel (2020). What explains the relationship between spatial and mathematical skills? A review of evidence from brain and behavior. Psychonomic Bulletin & Review.

[B17-jintelligence-13-00030] Heil Martin, Jansen-Osmann Petra (2008). Sex differences in mental rotation with polygons of different complexity: Do men utilize holistic processes whereas women prefer piecemeal ones?. Quarterly Journal of Experimental Psychology.

[B18-jintelligence-13-00030] Holmqvist Kenneth, Nyström Marcus, Andersson Richard, Dewhurst Richard, Jarodzka Halszka, Weijer Joost Van de (2011). Eye Tracking: A Comprehensive Guide to Methods and Measures.

[B19-jintelligence-13-00030] Höffler Tim N. (2010). Spatial ability: Its influence on learning with visualizations—A meta-analytic review. Educational Psychology Review.

[B20-jintelligence-13-00030] Ihaka Ross, Gentleman Rober (1996). R: A language for data analysis and graphics. Journal of Computational and Graphical Statistics.

[B21-jintelligence-13-00030] Jäkel Frank, Singh Manish, Wichmann Felix A., Herzog Michael. H. (2016). An overview of quantitative approaches in Gestalt perception. Vision Research.

[B22-jintelligence-13-00030] Just Marcel A., Carpenter Patricia A. (1976). Eye fixations and cognitive processes. Cognitive Psychology.

[B23-jintelligence-13-00030] Kagan Jerome (1965). Reflection-impulsivity and reading ability in primary grade children. Child Development.

[B24-jintelligence-13-00030] Kail Rober, Carter Philip, Pellegrino James (1979). The locus of sex differences in spatial ability. Perception & Psychophysics.

[B25-jintelligence-13-00030] Kanamori Nobuhiro, Yagi Akihiro (2002). The difference between flipping strategy and spinning strategy in mental rotation. Perception.

[B26-jintelligence-13-00030] Kirschner Paul A. (2017). Stop propagating the learning styles myth. Computers & Education.

[B27-jintelligence-13-00030] Kozhevnikov Maria, Hegarty Mary, Mayer Richard E. (2002). Revising the visualizer-verbalizer dimension: Evidence for two types of visualizers. Cognition and Instruction.

[B28-jintelligence-13-00030] Kyllonen Patrick C., Christal Raymond E. (1990). Reasoning ability is (little more than) working-memory capacity?. Intelligence.

[B29-jintelligence-13-00030] Lam Chi-Ming (2007). Is Popper’s falsificationist heuristic a helpful resource for developing critical thinking?. Educational Philosophy and Theory.

[B30-jintelligence-13-00030] Linn Marcia C., Petersen Anne C. (1985). Emergence and Characterization of Sex Differences in Spatial Ability: A Meta-Analysis. Child Development.

[B31-jintelligence-13-00030] Liu Yaohui, Zhan Peida, Fu Yanbin, Chen Qipeng, Man Kaiwen, Luo Yikun (2023). Using a multi-strategy eye-tracking psychometric model to measure intelligence and identify cognitive strategy in Raven’s advanced progressive matrices. Intelligence.

[B32-jintelligence-13-00030] Lohman David. F. (1979). Spatial Ability: A Review and Reanalysis of the Correlational Literature.

[B33-jintelligence-13-00030] Lohman David. F. (1996). Spatial Ability and g. Human Abilities.

[B34-jintelligence-13-00030] Man Kaiwen, Harring Jeffrey R. (2019). Negative binomial models for visual fixation counts on test items. Educational and Psychological Measurement.

[B35-jintelligence-13-00030] Man Kaiwen, Harring Jeffrey R., Zhan Peida (2022). Bridging models of biometric and psychometric assessment: A three-way joint modeling approach of item responses, response times, and gaze fixation counts. Applied Psychological Measurement.

[B36-jintelligence-13-00030] Maris Eric (1999). Estimating multiple classification latent class models. Psychometrika.

[B37-jintelligence-13-00030] McGee Mark G. (1979). Human spatial abilities: Psychometric studies and environmental, genetic, hormonal, and neurological influences. Psychological Bulletin.

[B38-jintelligence-13-00030] Mitchell Tom M., Utgoff Paul E., Banerji Ranan (1983). Learning by experimentation: Acquiring and refining problem-solving heuristics. Machine Learning: An Artificial Intelligence Approach.

[B39-jintelligence-13-00030] Miyake Akira, Shah Priti (1999). Models of Working Memory.

[B40-jintelligence-13-00030] Miyake Akira, Friedman Naomi P., Rettinger David A., Shah Priti, Hegarty Mary (2001). How are visuospatial working memory, executive functioning, and spatial abilities related? A latent-variable analysis. Journal of Experimental Psychology: General.

[B41-jintelligence-13-00030] Nazareth Alina, Killick Rebecca, Dick Anthony S., Pruden Shannon M. (2019). Strategy selection versus flexibility: Using eye-trackers to investigate strategy use during mental rotation. Journal of Experimental Psychology: Learning, Memory, and Cognition.

[B42-jintelligence-13-00030] Nickerson Raymond S. (1998). Confirmation bias: A ubiquitous phenomenon in many guises. Review of General Psychology.

[B43-jintelligence-13-00030] Nuttall Ronald L., Casey M. Beth, Pezaris Elizabeth (2005). Spatial Ability as a Mediator of Gender Differences on Mathematics Tests: A Biological-Environmental Framework.

[B44-jintelligence-13-00030] Pellegrino James W., Mumaw Randall J., Shute Valerie J. (1985). Analyses of spatial aptitude and expertise. Test Design.

[B45-jintelligence-13-00030] Petrova Kate, Gross James J. (2023). The future of emotion regulation research: Broadening our field of view. Affective Science.

[B46-jintelligence-13-00030] Plummer Martyn (2017). JAGS Version 4.3. 0 User Manual. https://sourceforge.net/projects/mcmc-jags/files/Manuals/4.x/.

[B47-jintelligence-13-00030] Plummer Martyn (2023). JAGS: Just Another Gibbs Sampler. https://mcmc-jags.sourceforge.io.

[B48-jintelligence-13-00030] Plummer Martyn, Best Nicky, Cowles Kate, Vines Karen (2006). CODA: Convergence diagnosis and output analysis for MCMC. R News.

[B49-jintelligence-13-00030] Popper Karl R. (1963). Science as falsification. Conjectures and Refutations.

[B50-jintelligence-13-00030] Quasha William H., Likert Rensis (1937). The revised Minnesota paper form board test. Journal of Educational Psychology.

[B51-jintelligence-13-00030] Ramful Ajay, Lowrie Thomas, Logan Tracy (2017). Measurement of spatial ability: Construction and validation of the spatial reasoning instrument for middle school students. Journal of Psychoeducational Assessment.

[B52-jintelligence-13-00030] Reilly David, Neumann David L. (2013). Gender-role differences in spatial ability: A meta-analytic review. Sex Roles.

[B53-jintelligence-13-00030] Salhi Saïd (2017). Heuristic Search: The Emerging Science of Problem Solving.

[B54-jintelligence-13-00030] Serbin Lisa A., Zelkowitz Phyllis, Doyle Anna-Beth, Gold Dolores, Wheaton Blair (1990). The socialization of sex-differentiated skills and academic performance: A mediational model. Sex Roles.

[B55-jintelligence-13-00030] Shepard Roger N. (1978). The mental image. American Psychologist.

[B56-jintelligence-13-00030] Shepard Roger N., Metzler Jacqueline (1971). Mental rotation of three-dimensional objects. Science.

[B57-jintelligence-13-00030] Sternberg Robert J., Grigorenko Elena L. (1997). Are cognitive styles still in style?. American Psychologist.

[B58-jintelligence-13-00030] Sternberg Robert J., Kaufman Scott Barry (2011). The Cambridge Handbook of Intelligence.

[B59-jintelligence-13-00030] Thurstone Louis Leon, Thurstone Thelma Gwinn (1941). Factorial studies of intelligence. Psychometric Monographs.

[B60-jintelligence-13-00030] Unkelbach Christian (2006). The learned interpretation of cognitive fluency. Psychological Science.

[B61-jintelligence-13-00030] Uttal David H., Meadow Nathaniel G., Tipton Elizabeth, Hand Linda L., Alden Alison R., Warren Christopher, Newcombe Nora S. (2013). The malleability of spatial skills: A meta-analysis of training studies. Psychological Bulletin.

[B62-jintelligence-13-00030] Van der Linden Wim J. (2006). A lognormal model for response times on test items. Journal of Educational and Behavioral Statistics.

[B63-jintelligence-13-00030] Venables W. N., Smith D. M. (2003). The R development core team. An Introduction to R.

[B64-jintelligence-13-00030] Wagemans Johan, Feldman Jacob, Gepshtein Sergei, Kimchi Ruth, Pomerantz James R., Helm Peter A. Van der, Leeuwen Cees Van (2012). A century of Gestalt psychology in visual perception: II. Conceptual and theoretical foundations. Psychological Bulletin.

[B65-jintelligence-13-00030] Wai Jonathan (2022). Spatial thinkers receive their due. Science.

[B66-jintelligence-13-00030] Wai Jonathan, Worrell Frank C. (2016). Helping disadvantaged and spatially talented students fulfill their potential: Related and neglected national resources. Policy Insights from the Behavioral and Brain Sciences.

[B67-jintelligence-13-00030] Wai Jonathan, Lubinski David, Benbow Camilla P. (2009). Spatial ability for STEM domains: Aligning over 50 years of cumulative psychological knowledge solidifies its importance. Journal of Educational Psychology.

[B68-jintelligence-13-00030] Wang Yingxu, Chiew Vincent (2010). On the cognitive process of human problem solving. Cognitive Systems Research.

[B69-jintelligence-13-00030] Yilmaz Işık (2009). Landslide susceptibility mapping using frequency ratio, logistic regression, artificial neural networks and their comparison: A case study from Kat landslides (Tokat—Turkey). Computers & Geosciences.

[B70-jintelligence-13-00030] Zhan Peida, Jiao Hong, Liao Dandan (2018). Cognitive diagnosis modelling incorporating item response times. British Journal of Mathematical and Statistical Psychology.

[B71-jintelligence-13-00030] Zhan Peida, Jiao Hong, Man Kaiwen, Wang Lijun (2019). Using JAGS for Bayesian cognitive diagnosis modeling: A tutorial. Journal of Educational and Behavioral Statistics.

[B72-jintelligence-13-00030] Zhan Peida, Man Kaiwen, Wind Stefanie A., Malone Jonathan (2022). Cognitive diagnosis modeling incorporating response times and fixation counts: Providing comprehensive feedback and accurate diagnosis. Journal of Educational and Behavioral Statistics.

